# Supplementary material for: Nanofaceting as a stamp for periodic graphene charge carrier modulations
Source: Sci Rep. 2016 Apr 4;6:23663. doi: 10.1038/srep23663 (PMC4819194; doi:10.1038/srep23663)
Supplement: Supplementary Information [file srep23663-s1.pdf]

Supplementary: Nanofaceting as a stamp for periodic graphene charge carrier modulations

M. Vondráček, M. Kučera, L. Fekete, J. Kopeček, J. Lančok, and J. Honolka

Institute of Physics of the Czech Academy of Sciences,

Na Slovance 2, CZ-182 21 Praha 8, Czech Republic

D. Kalita, J. Coraux, and V. Bouchiat

Univ. Grenoble Alpes, Inst. NEEL, F-38000 Grenoble, France and

Département Nanosciences, CNRS, Institut Néel, F-38042 Grenoble, France

Energy-dependent LEED

Fig. 1 summarizes typical patterns visible in energy-dependent LEED. The figure is a superposition of the LEED images acquired at different energies between 43 eV and 60 eV. Position-dependent LEED showed that these patterns are quite homogeneous over millimeter scales on the copper foil. Three energy-independent specular reflections define the three facet normals n_1, n_2, and n_3 aligned perpendicular to the $[1\bar{1}0]$ direction (intensity maxima at the centers of the specular reflections are cut for better visibility of remaining LEED pattern). They correspond to (111), (110), and $(22\bar{1})$, respectively, where (111) has by far the largest intensity and thus dominates the surface area. The assignment of crystal orientations to the specular reflections was done according to the distance of the three specular spots n_1, n_2, and n_3 on the LEED screen. Knowing the geometry of the LEED instrument the relative inclination angles with respect to $n_1 \parallel$ Cu[111] can be estimated with good accuracy.

Graphene spots are found on the distorted circles around the (111) and (110) facets, which move towards the respective specular reflections with increasing energy. For the $(22\bar{1})$ facet only a few spots are faintly visible (see arrows). On the circles around (111) and (110) the same three rotational graphene domains are visible, corresponding to relative angles $(0 \pm 2)^\circ$, $(+8 \pm 2)^\circ$, and $(+20 \pm 2)^\circ$ as indicated. A 4th domain is faintly visible at $(-8 \pm 1)^\circ$ especially on the high intensity (111) facet.

Additionally to the graphene spots, a p(2×2) superlattice is visible, 30° rotated with respect to the Cu(111) reciprocal lattice (see red unit cell vectors in the LEED image), which corresponds to the oxygen pattern reported recently by Gottardi et al. in 2015 (see Fig. 2, in Ref.[1]). The oxygen (2×2) superlattice spots move towards the (111) facet with increasing energy as expected.

Core level X-ray photoemission spectroscopy

Independently of the LEED studies, the presence of oxygen is directly evident from XPS data summarized in Fig. 2. Fig. 2a shows XPS scans over a wide energy range for regions with (red circle) and without (white circle) graphene. C 1s core level spectra in panel Fig. 2b reveal a sharp carbon peak on graphene, which is much larger in intensity compared

to that on the bare Cu foil, and is located at a higher binding energy $E_{\mathrm{B}} \approx 284.7\,\mathrm{eV}$ typical for graphene[2]. Oxygen O $1s$ spectra presented in Fig. 2c evidence higher oxygen amounts on bare Cu foil and less on the graphene covered areas. Partly oxidized surfaces underneath graphene are expected from the observed oxygen $\mathrm{p}(2 \times 2)$ superlattice on Cu(111) in LEED. Copper oxides with O $1s$ core levels at $E_{\mathrm{B}} \sim 531\,\mathrm{eV}$ represent only minor contributions, whereas the main oxygen peak at $E_{\mathrm{B}} \sim 533\,\mathrm{eV}$ can be mostly attributed to adsorbed H_2O and to lesser extend to any C-O related bonds[2,3]. Comparing Cu $3p$ and $2p_{3/2}$ core level XPS spectra in Fig. 2d-e to the literature, they resemble those of clean copper.

Derivation of carrier modulations

Fig. 3 shows the dispersion relations of facet n_3 at the high-symmetry k space points K and K' as an energy cut along K − K' (see inset on the bottom right). The Fermi level E_{F} is defined by the disappearance of k-PEEM intensity as shown in a typical intensity profile in green. Linear fits of the dispersion are drawn, which correspond to slopes of 6.3 eVÅ. Using the graphene relation $E(k) = \hbar v_{\mathrm{F}} \cdot k$, we derive a Fermi velocity $v_{\mathrm{F}} = (0.95 \pm 0.05) \times 10^6\,\mathrm{m/s}$. In the energy range $\Delta E = (E_{\mathrm{DP}} - E)$ from the Dirac point, where the linear relation holds, the Fermi velocity determines the density of states $D(\Delta E) = D_0 \cdot |\Delta E|$ at the K-points via the following relation[4]:

$$D_0 = \frac{2A_c}{\pi(\hbar v_{\mathrm{F}})^2} \tag{1}$$

Hereby, A_c is the graphene unit cell area of $0.051\,\mathrm{nm}^2$. With $v_{\mathrm{F}} = (0.95 \pm 0.05) \times 10^6\,\mathrm{m/s}$ we get $D_0 = (0.085 \pm 0.009)$ per (eV^2 and graphene unit cell area), a value similar to the one found e.g. by Giovannetti et al.[5] for graphene on Pt(111).

A simple integration of $D(\Delta E)$ over energy finally gives the number of transferred carriers per unit cell area:

$$n(\Delta E) = 0.085/\mathrm{eV}^2 \cdot (\Delta E)^2/2 \tag{2}$$

Since within the error bar the Fermi velocity was found the same for all facets n_1, n_2, and n_3 this relation can be used in all three cases. Scaling the area to $1\,\mathrm{cm}^2$ the respective doping levels $\Delta E = (0.44 \pm 0.10), (0.63 \pm 0.10)$, and $(0.82 \pm 0.10)\,\mathrm{eV}$ correspond to carrier densities of $n = 1.6 \times 10^{13}\,\mathrm{cm}^{-2}$, $3.3 \times 10^{13}\,\mathrm{cm}^{-2}$, and $5.7 \times 10^{13}\,\mathrm{cm}^{-2}$, respectively. We estimate the according relative errors to 60%, 45%, and 35%. These errors are not to be added when

calculating relative changes Δn.

Dark-field microscopy statistics

In Fig. 4 additional dark field measurements on further example islands are presented. Fig. 4a shows a small island of about 15 μm width, which is single domain as visible in the missing dark field contrast in the middle column. Small islands generally were found in single domain states. For the larger islands Fig. 4(b-d) on the other hand the formation of triangular shaped domain seeds are visible. Island shapes and seed geometries become less symmetric and more corrugated with increasing island sizes. The bright field images after sputtering (rightmost column) reveal a sudden change of the micrometer scale roof-top modulation of the underlying Cu at the domain boundaries, almost always towards lager periodicities.

It is worth to note that large islands occasionally host patches of 2nd and 3rd layer graphene (see Fig. 4c and d). They can be straightforwardly identified in energy-filtered PEEM, where multiple graphene layer areas appear darker due to the change in work function contrast. Interestingly, the growth of the 2nd layer changes the roof-top modulation in a similar way as the triangular shaped rotational domain seeds within single layer graphene. Also for 2nd layer areas the periodicity of the roof-top structure is larger. Indeed, this supports recent reports of the 2nd layer of graphene growing below the 1st layer[6], where it can restructure the copper surface by direct contact. Unfortunately, the 2nd layer graphene orientation φ could not be identified without doubt.

[1] S. Gottardi, K. Müller, L. Bignardi, J. C. Moreno-López, T. A. Pham, O. Ivashenko, M. Yablonskikh, A. Barinov, J. Björk, P. Rudolf, and M. Stöhr, Nano Letters **15**, 917 (2015).

[2] R. Blume, P. R. Kidambi, B. C. Bayer, R. S. Weatherup, Z.-J. Wang, G. Weinberg, M.-G. Willinger, M. Greiner, S. Hofmann, A. Knop-Gericke, and R. Schlogl, Phys. Chem. Chem. Phys. **16**, 25989 (2014).

[3] S. Yamamoto, H. Bluhm, K. Andersson, G. Ketteler, H. Ogasawara, M. Salmeron, and A. Nilsson, Journal of Physics: Condensed Matter **20**, 184025 (2008).

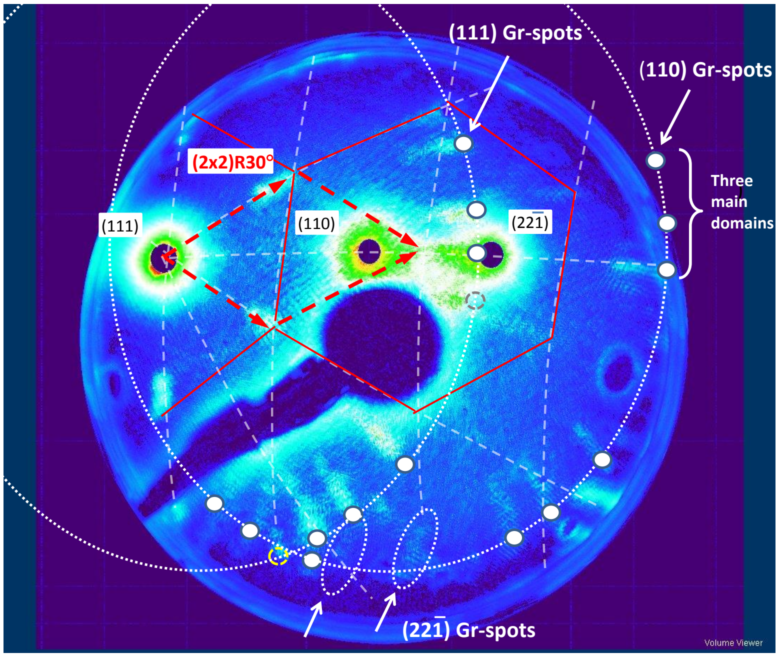

FIG. 1. **Energy dependent LEED analysis.** The energy range is between 43 eV and 60 eV. For better visibility the peak intensity of the dominant specular spots corresponding to (111), (110), and (22$\bar{1}$) facets is cut. Graphene spots are indicated as white dots located on dashed circles. The oxygen (2 × 2) hexagonal superlattice on (111) is shown in red including unit cell vectors. All diffraction spots move towards their respective specular spot with increasing energy.

[4] A. H. Castro Neto, F. Guinea, N. M. R. Peres, K. S. Novoselov, and A. K. Geim, Rev. Mod. Phys. **81**, 109 (2009).

[5] G. Giovannetti, P. A. Khomyakov, G. Brocks, V. M. Karpan, J. van den Brink, and P. J. Kelly, Phys. Rev. Lett. **101**, 026803 (2008).

[6] S. Nie, W. Wu, S. Xing, Q. Yu, J. Bao, S. shem Pei, and K. F. McCarty, New Journal of Physics **14**, 093028 (2012).

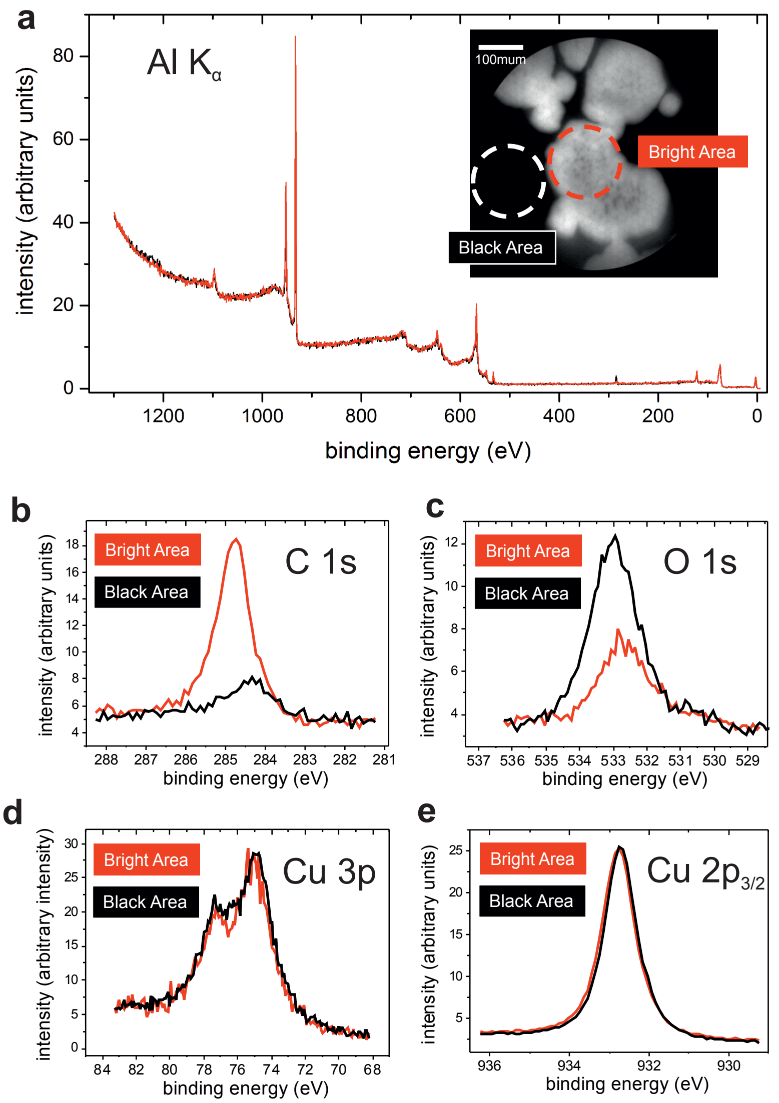

FIG. 2. **X-ray photoemission spectroscopy.** Comparison of XPS core level measurements on a large graphene patch (bright area) and on the bare copper foil (dark area). **(a)** Wide XPS scan. **(b)** and **(c)**: C 1s and O 1s XPS spectra. **(d)** and **(e)** Cu 3p and 2$p_{3/2}$ XPS spectra.

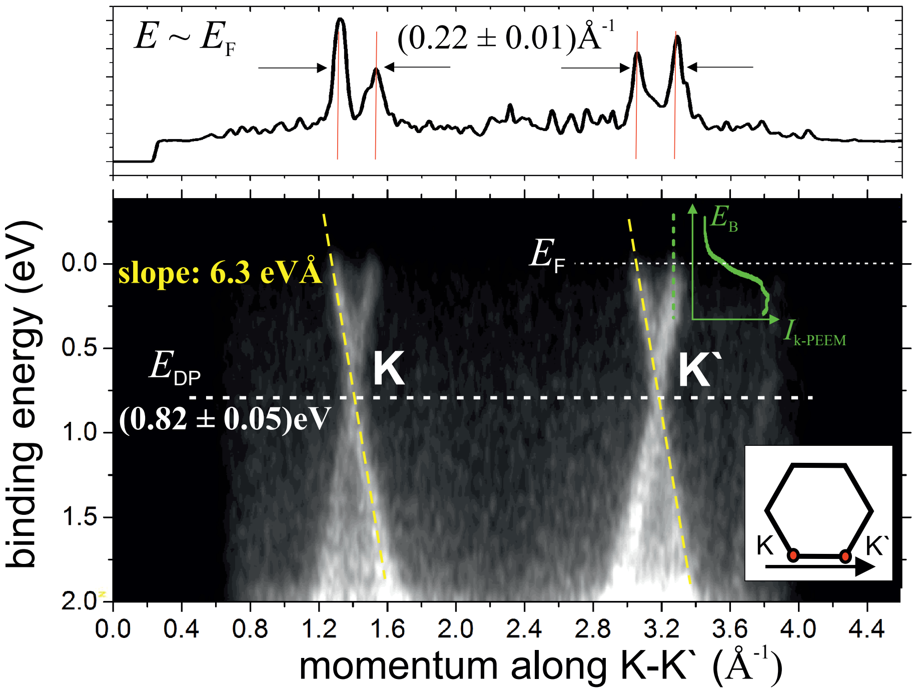

FIG. 3. **Fermi velocity fit.** Measured dispersion relations of facet n_3 at the high-symmetry k space points K and K' as an energy cut along K − K'. The direction of the cut is sketched in the inset on the bottom right. For n_3 the Dirac point is found at $E_{\mathrm{DP}} = (0.82 \pm 0.05)\,\mathrm{eV}$.

The Fermi level E_{F} is defined by the disappearance of k-PEEM intensity. A typical vertical intensity profile $I_{\text{k-PEEM}}$ along the dashed green line is plotted on the top right side, which defines zero binding energy. At the top panel a horizontal intensity profile along K − K' is plotted at approximately E_{F}.

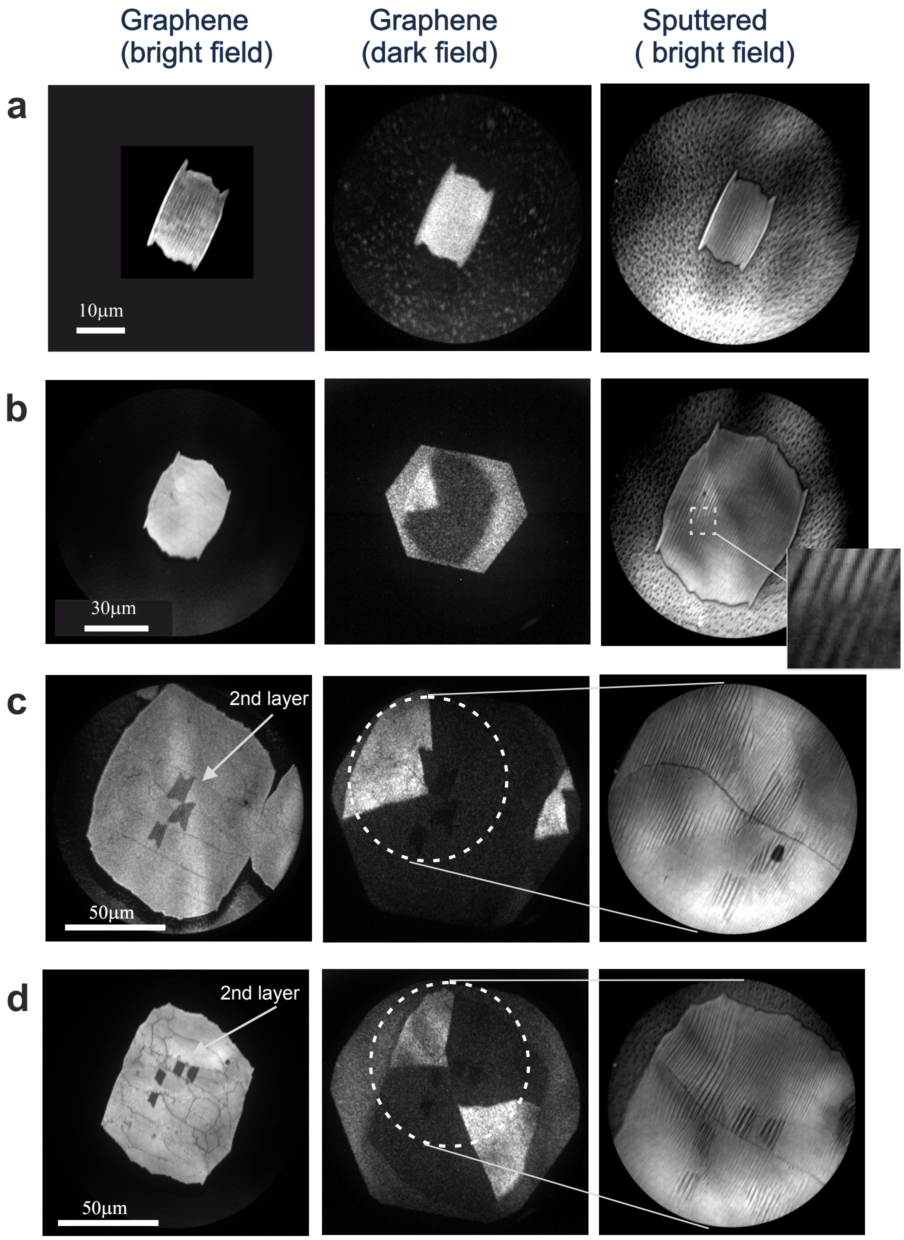

FIG. 4. **Bright field and dark field PEEM images of different graphene islands.** Images before (left and middle column) and after mild sputtering (right column) are shown. If right columns show zoom-ins, the respective area is indicated as circles or squares in the middle column. **(a)** small single-domain island without DF contrast and with homogeneous stripe structure, **(b)** small island with rotational domain and non-homogeneous stripe structure visible also after sputtering, **(c)**- **(d)** larger coalescing islands with dark field contrast. 2nd layer graphene areas are visible as darker patches due to the work function contrast in energy filtered bright field PEEM.
